# Gendered analysis of care work burden and mental health using data from the Gutenberg Covid-19 study

**DOI:** 10.1038/s41598-025-11841-x

**Published:** 2025-07-22

**Authors:** E. Engwicht, J. Petersen, E. Braehler, F. Wicke, J. Koenig, T. Muenzel, A. K. Schuster, K. Lackner, P. S. Wild, M. E. Beutel, Nora Hettich-Damm

**Affiliations:** 1 Department of Psychosomatic Medicine and Psychotherapy, University Medical Center Mainz, Langenbeckstraße 1, 55131 Mainz, Germany; 2 Department of Psychiatry and Psychotherapy, Leipzig University Medical Center, Liebigstraße 18, 04103 Leipzig, Germany; 3 Pediatric Epidemiology, Institute of Medical Biostatistics, Epidemiology and Informatics, University Medical Center Mainz, Langenbeckstraße 1, 55131 Mainz, Germany; 4 Center for Cardiology, University Medical Center Mainz, Langenbeckstraße 1, 55131 Mainz, Germany; 5 Department of Ophthalmology, University Medical Center Mainz, Langenbeckstraße 1, 55131 Mainz, Germany; 6 Institute for Clinical Chemistry and Laboratory Medicine, University Medical Center Mainz, Langenbeckstraße 1, 55131 Mainz, Germany; 7 Preventive Cardiology and Preventive Medicine, Center for Cardiology, University Medical Center Mainz, Langenbeckstraße 1, 55131 Mainz, Germany; 8 German Center for Cardiovascular Research (DZHK), Partner Site Rhine-Main, Potsdamer Str. 58, 10785 Berlin, Germany; 9 Department of Psychosomatic Medicine and Psychotherapy, University Medical Center Mainz, Untere Zahlbacher, Straße 8, 55131 Mainz, Germany

**Keywords:** Gender equity, Mental health, Care work, Employment, COVID-19 pandemic, Somatization, Anxiety, Depressiveness, Psychology, Risk factors

## Abstract

In light of the growing awareness of the unequal distribution of care work, this study aimed to analyze gender differences in burdens of care work and associations with mental health during the COVID-19 pandemic, with a focus on employment status. Therefore, data from the population-representative Gutenberg COVID-19 cohort, collected between October 2020 and April 2021, in the city of Mainz and the County of Mainz-Bingen, Germany, were used. Out of the whole cohort, people living with children in the household were included (*N* = 2,043). Feeling burdened by care work and mental health outcomes were assessed by validated self-report questionnaires. Descriptive analyses and multilinear regression analyses were performed. Results showed that feeling burdened by care work was more likely for women than for men. For men, feeling burdened by care work was significantly associated with depressiveness, anxiety, and somatization. For women, difficulties with child raising were associated with depressiveness. Part-time working men and women did not differ with respect to mental health or care work burden, whereas unemployed and full-time working women showed significantly worse mental health and reported more burden of care than unemployed and full-time working men. Hence, this study showed gender inequalities. For women, worsening external conditions for childcare appeared to be a risk factor. Men with care work responsibilities seem to benefit, concerning their mental health, from full-time paid work. The data underscore the importance of mitigating the burden of care work, especially to improve conditions for women and part-time workers.

## Introduction

Previous studies already pointed out the pre-pandemic existing poorer mental health of women compared to men as well as the unequal costs of the pandemic for women’s mental health^1–3^. The present study highlights the issue of caregiving burdens as one of many potentially underlying factors. The gender care gap and its consequences for individuals (e.g. old-age poverty) on the one hand and society on the other hand (e.g. skills shortage) have increasingly become a focus of public interest. Already before the COVID-19 pandemic women were more likely to take over unpaid care work responsibilities than men^4^. Additionally, existing data during the pandemic indicated that women primarily took on additional task related to care work^5,6^. Studies addressing the effect of increased care work responsibilities for mental health from a gender-specific point of view showed that this was at the expense of female caregivers’ mental health: Data from Japan showed that during school closures, especially low-educated women with children who attended primary school reported worse mental health^7^. Research from South Africa showed an association between depressiveness and hours spent caring for children^8^. An analysis from the US showed that mothers from the US were strongly affected due to emotional strain^9^. Apart from these findings, which indicate that mainly women with care work responsibility suffered from worse mental health during the pandemic, data from Canada collected online between May 2020 and March 2021 showed a stronger association with anxiety symptoms among men living with children compared to women living with children^10^.

Concerning risk and protective factors for reduced mental health of caregivers during the pandemic, researchers from Australia examined data from parents of children between 0 and 18 years. They pointed out a strong association between loneliness and psychological distress, as well as a significant association between higher social support and reduced psychological distress. Concerning resilience, no significant association was found^11^. Regarding potential benefits of care work during the pandemic, such as more time with family members or greater appreciation of domestic tasks, and their significance for psychological well-being, the current data situation is poor as previous studies have mainly focused on risk factors. A qualitative survey reported that the majority of parents did not identify out any positive aspects. Less than 15% of participants mentioned more time with family members as a positive aspect of the pandemic^12^.

The interplay between unpaid care work tasks and the amount of paid work is also of interest with respect to the mental health of male and female caregivers during the pandemic: Data from an online survey conducted in the United Kingdom during lockdown reported higher psychological distress among fathers who had reduced their working hours as the only member of the household. This effect was not observed among mothers^13^. Pre-pandemic data from the German Health Study reported a lower degree of depressive symptoms among fathers working full-time compared to fathers with non-full-time employment, while this observation was not made among women^14^. The struggle of combining care work and paid work tasks and its costs for mental health was examined by Portuguese researchers, who found a positive correlation between impaired mental health and work-family-personal-time conflict for women and men^15^.

In Addition to existing studies based primarily online surveys with convenience samples, the current study analyzed data from a face-to-face cohort study with additional questionnaires using a representative sample of more than 2,000 individuals living with children under 18 years of age. In contrast to the mentioned studies, which inferred burdens of care work based on the extent of time for childcare or adjustment of working hours, this study aimed to directly assess the perceived burdens of care work from a gender-specific persprective. Moreover, the current study includes the analysis of possible benefits of care work during the pandemic as possible moderators of the mental health of parents as well as the role of the amount of paid work. The study evaluates the following questions and hypotheses:

1. Which sample characteristics distinguish participants living with children in the household who feel burdened by care work from those who do not feel burdened?
2. How is the burden of care work associated with reduced mental health regarding depressiveness, anxiety, and somatization?

- The effect of care work burdens on mental health is stronger for women than for men living with children in the household.
- The benefits of care work (more time with family members, greater appreciation of domestic services) moderate the association between care work burdens and the mental health of people living with children.

3) Does the mental health and experienced burden of care differ between men and woman with regard to their employment status?

## Method

### Study design and sample

Our data are drawn from the population-representative Gutenberg COVID-19 Study (GCS). After the outbreak of SARS-CoV-2, the respondents of the Gutenberg Health Study (GHS^16^) – a large-scale population-based cohort study – were invited to participate in the GCS. The overall objective of the GCS is to comprehensively and systematically investigate the epidemiology of the COVID-19 pandemic in the population.

The GCS sample consists of *N* = 8,121 participants of the GHS and *N* = 2,129 individuals additionally recruited between the ages of 25 and 44 (young cohort). In total, the GCS cohort includes 10,250 individuals aged 25 to 88 years. The sample was stratified by gender, age, and place of residence (city of Mainz / County of Mainz-Bingen). Individuals who were mentally or physically unable to visit the study center as well as individuals with low proficiency in the German language were excluded from the study. For the GCS, two visits to the study center took place, during which a computer-assisted personal interview and sequential sampling of biomaterial were performed. Questionnaires were sent prior to the visit to the study site. For the present study, we included respondents who participated in the data collection from October 2020 to April 2021 and who lived with children and youths under the age of 18. This left us with a sample of *N* = 2,043 individuals.

The requirements of Good Clinical Practice (GCP), Good Epidemiological Practice (GEP), and the ethical standards of the Declaration of Helsinki were fulfilled during the study’s design, implementation, and analysis. Furthermore, the Federal Data Protection Act’s requirements were adhered to. The Ethics Committee (Ethics Committee of the Rhineland-Palatinate Medical Association, reference number 2018–13720_4), as well as the Data Protection Officer of the Johannes Gutenberg University Hospital Mainz, assessed all study-relevant documentation for the Gutenberg Health Study and the Gutenberg COVID-19 Study and gave a positive vote. The Data Protection Commissioner of Rhineland-Palatinate approved the drawing of the sample via the citizens’ registration offices. Written informed consent was obtained from all subjects.

### Measures

The care burden prevalence was assessed using an item from the PHQ-Stress (Patient Health Questionnaire) that asked how much a respondent felt burdened by the care of children, parents, or other family members during the past four weeks on a three-point scale (0 = ‘not burdened at all’ to 2 = ‘very burdened’). We chose this item because it is part of a firmly established questionnaire and the care work burden was our main focus. This item was recoded dichotomously for most of the analysis (except for the group mean comparisons) with the cut-off set at ≥ 1.

Positive childcare variables included the two self-constructed variables “*Which of the following aspects of the pandemic did you experience positively? (1) Spending more time with children or close relatives*,* (2) experiencing higher appreciation for domestic work (e.g.*,* household chores*,* childcare or care of a relative)”.* Additionally, we collected data on “*Which of the following aspects of the pandemic did you experience? (1) Experiencing difficulties with childcare and child raising (e.g.*,* restricted care or schooling)”.* Those three variables were assessed using a 4-point scale (1 = ‘does not apply at all’ to 4 = ‘applies fully’) and subsequently recoded dichotomously with the cut-off being at ≥ 3.

In order to measure depressiveness, we used the self-administered Patient Health Questionnaire (PHQ-9) depression scale^17–19^. The GAD-2 questionnaire^20–24^ was used to quantify anxiety. Somatization was assessed using the self-administered Somatic Symptom Scale (SSS-8)^25^. The PHQ-9 and GAD-2 questionnaires refer to psychological well-being during the past two weeks, and the SSS-8 to the past seven days. We used the three-item Loneliness Scale^26,27^which was shortened from the 20-item Revised UCLA Loneliness Scale (R-UCLA^28^), , to measure loneliness. Resilient coping was assessed using the Brief Resilient Coping Scale (BRCS)^29,30^. Social support was measured using the Brief Social Support Scale (BS-6)^31^. All items of each scale were summed up to a composite score.

Finally, we also assessed the sociodemographic variables gender, age, living with children (categorized into 0-6-, 7–14 and 15-17year-old children), partnership status (single, living apart from the partner, living with the partner), employment status (no current employment (respectively no paid work, including inter alia retirement and joblessness), part-time, full-time) and socioeconomic status^32^. The participant’s gender was registered based on the interviewer`s assignment. All measurement instruments were collected using a computer-assisted personal interview (CAPI).

### Statistical analysis

We first performed a descriptive analysis to identify sociodemographic differences between people who felt burdened by care work and those who did not and conducted group difference tests. We opted for chi-square tests and t-tests, with a p-value *p* < 0.05 indicating a significant discrepancy between the groups. We then repeated this step for men and women separately.

Next, we performed multilinear regression analysis cross-sectionally for depressiveness, anxiety, and somatization. All analyzing and testing was performed using R (R Version 4.0.3., R-Studio Version 1.3.1093, packages: car, carData, dplyr, psych, sandwich, jtools, lm.beta).

Finally, we performed group mean difference tests between the different employment status groups for depressiveness, anxiety, somatization, and care burden, separated by gender. Here, we also opted for t-tests to identify significant differences between the groups.

## Results

### Descriptive analyses

Table 1 shows the characteristics of the whole sample and the group comparisons between participants who reported being burdened by caring for children, parents, or other family members and individuals who reported not being burdened by these tasks. Furthermore, it shows the same comparisons separately for women and men. Individuals who reported being burdened were more often women, younger, had more frequently children under the age of six and less frequently children between 13 and 17, reported more often higher education, part-time or full-time employment, less appreciation for domestic work, more difficulties with childcare and child-raising, lower levels of social support and a smaller social network, lower scores in resilient coping, and higher scores in loneliness, depressiveness, anxiety, and somatization. The same applied to the comparison between women, with the exception that education, employment, and resilient coping did not differ between women who felt burdened and those who did not. In contrast to the results of the whole sample, men who felt burdened did not differ from those who did not with regard to employment, appreciation of domestic work, social network size, and resilient coping.

**Table 1:** Sample characteristics and group comparisons for burdens of care.

	Sample (*N* = 2,043)	All			Women			Men		
		Being burdened (*N* = 1,265)	Not being burdened (*N* = 778)	*p*	Being burdened (*N* = 734)	Not being burdened (*N* = 321)	*p*	Being burdened (*N* = 531)	Not being burdened (*N* = 457)	*p*
Gender *N* = 2043										
male	988 (48.4%)	531 (42.0%)	457 (58.7%)	**0.000**						
female	1055 (51.6%)	734 (58.0%)	321 (41.3%)	**0.000**						
Age (mean) *N* = 2043	44.84 (8.98)	43.53 (7.94)	46.97 (10.06)	**0.000**	42.49 (7.66)	45.49 (9.52)	**0.000**	44.96 (8.18)	48.10 (10.31)	**0.000**
Migration background (yes) *N* = 2039	424 (20.8%)	278 (22.0%)	146 (18.8%)	0.090	166 (22.6%)	55 (17.1%)	0.052	112 (21.2%)	91 (20.0%)	0.695
Partnership *N* = 1764										
single	78 (4.4%)	57 (5.1%)	21 (3.2%)	0.075	45 (6.9%)	16 (5.7%)	0.607	12 (2.6%)	5 (1.3%)	0.289
living apart	52 (3.0%)	34 (3.1%)	18 (2.8%)	0.820	26 (4.0%)	8 (2.9%)	0.519	8 (1.8%)	10 (2.7%)	0.503
living together	1634 (92.6%)	1019 (91.8%)	615 (94.0%)	0.101	581 (89.1%)	255 (91.4%)	0.348	438 (95.6%)	360 (96.0%)	0.929
Living with children *N* = 2043										
under 6	959 (46.9%)	660 (52.2%)	299 (38.4%)	**0.000**	377 (51.3%)	113 (35.2%)	**0.000**	283 (53.3%)	186 (40.7%)	**0.000**
7 to 14	763 (37.4%)	466 (36.8%)	297 (38.2%)	0.576	275 (37.5%)	128 (39.9%)	0.501	191 (36.0%)	169 (37.0%)	0.793
15 to 17	321 (15.7%)	139 (11.0%)	182 (23.4%)	**0.000**	82 (11.2%)	80 (24.9%)	**0.000**	57 (10.7%)	102 (22.3%)	**0.000**
Number of children (mean) *N* = 1660	1.85 (1.17)	1.85 (1.05)	1.84 (1.35)	0.840	1.83 (0.84)	1.70 (0.97)	0.059	1.89 (1.29)	1.93 (1.54)	0.660
SES	16.58 (3.51)	16.71 (3.45)	16.38 (3,59)	0.047	16.47 (3.47)	16.12 (3.42)	0.137	17.04 (3.38)	16.57 (3.70)	0.042
SES Education	5.31 (1.82)	5.45 (1,76)	5.08 (1.90)	0.000	5.43 (1.74)	5.08 (1.82)	0.004	5.49 (1.79)	5.09 (1.95)	0.001
SES Income	6.03 (1.22)	6.00 (1,25)	6.08 (1.16)	0.125	5.92 (1.34)	6.04 (1.21)	0.163	6.10 (1.11)	6.11 (1.13)	0.919
SES Occupation	5.24 (1.33)	5.26 (1.31)	5.22 (1.35)	0.488	5.12 (1.27)	5.01 (1.29)	0.192	5.45 (1.34)	5.36 (1.38)	0.310
Tertiary Education (yes) *N* = 1301	915 (70.3%)	545 (73.8%)	370 (65.7%)	**0.002**	279 (71.5%)	146 (67.6%)	0.356	266 (76.4%)	224 (64.6%)	**0.001**
Employment *N* = 1995										
unemployed	191 (9.6%)	114 (9.2%)	77 (10.1%)	0.548	93 (13.0%)	49 (15.7%)	0.288	21 (4.0%)	28 (6.3%)	0.154
part-time	742 (37.2%)	520 (42.1%)	222 (29.3%)	**0.000**	464 (65.8%)	188 (60.3%)	0.186	56 (10.8%)	34 (7.6%)	0.115
fulltime	1062 (53.2%)	602 (48.7%)	460 (60.6%)	**0.000**	159 (22.2%)	75 (24.0%)	0.573	443 (85.2%)	385 (86.1%)	0.747
Benefits of care work										
Higher appreciation of domestic services *N* = 2011	2.29 (0.83)	2.25 (0.83)	2.37 (0.84)	**0.002**	2.14 (0.83)	2.27 (0.81)	**0.019**	2.41 (0.79)	2.44 (0.84)	0.599
More time with children or family members *N* = 2018	2.96 (0.90)	2.96 (0.88)	2.96 (0.92)	0.988	3.00 (0.88)	3.08 (0.87)	0.188	2.90 (0.88)	2.88 (0.95)	0.664
Burdens of care work										
Difficulties with childcare and raising children *N* = 2018	2.49 (1.76)	3.13 (1.61)	1.48 (1.49)	**0.000**	3.15 (1.66)	1.38 (1.50)	**0.000**	3.09 (1.54)	1.54 (1.49)	**0.000**
Social Support *N* = 1974	15.28 (3.17)	14.82 (3.28)	16.04 (2.81)	**0.000**	14.57 (3.40)	16.05 (2.87)	**0.000**	15.16 (3.08)	16.04 (2.77)	**0.000**
Social Network Size *N* = 2021	3.68 (0.56)	3.64 (0.59)	3.75 (0.51)	**0.000**	3.62 (0.60)	3.78 (0.47)	**0.000**	3.67 (0.57)	3.73 (0.54)	0.066
Resilient Coping (mean) *N* = 1958	10.97 (2.92)	10.82 (2.84)	11.22 (3.03)	**0.004**	10.61 (3.01)	10.89 (3.21)	0.191	11.12 (2.56)	11.45 (2.87)	0.060
Loneliness (mean) *N* = 1936	3.90 (2.53)	4.43 (2.58)	3.02 (2.17)	**0.000**	4.81 (2.64)	3.36 (2.07)	**0.000**	3.90 (2.41)	2.78 (2.20)	**0.000**
Depressiveness *N* = 1964	4.80 (4.04)	5.78 (4.28)	3.22 (2.99)	**0.000**	6.40 (4.47)	3.74 (2.98)	**0.000**	4.92 (3.85)	2.85 (2.95)	**0.000**
Anxiety *N* = 1774	0.90 (1.22)	1.13 (1.33)	0.49 (0.85)	**0.000**	1.31 (1.39)	0.62 (0.90)	**0.000**	0.87 (1.19)	0.39 (0.80)	**0.000**
Somatization *N* = 1973	5.93 (4.85)	6.85 (5.14)	4.44 (3.92)	**0.000**	7.71 (5.43)	5.45 (4.25)	**0.000**	5.67 (4.44)	3.72 (3.50)	**0.000**

Significant values (*p* < 0.05) are in bold.

### Associations between burdens of care work and decreased mental health

As shown in Table 2, cross-sectional regression analyses with mental health outcomes were performed for women and men separately. With respect to women, living with children under six years of age in the household was negatively and difficulties with childcare and raising children positively associated with depressiveness. Predictors explained 17.6% of the variance of somatization, 24% of anxiety and 31% of depressiveness.

Regarding results for men, feeling burdened by care work was significantly associated with depressiveness, anxiety, and somatization. Moderation analyses showed that the effect of care work burdens on anxiety and somatization was moderated by spending more time with family members. Predictors explained 17.1% of the variance of somatization, 21.4% of anxiety, and 27.8% of depressiveness.

The other predictors did not yield significant results. In the analyses, we controlled for age, SES (education, income, occupation), partnership, social support, social network size, resilient coping, loneliness, and employment status.

**Table 2:** Cross-sectional linear regression analyses of depressiveness, anxiety, and somatization on various aspects of burdens of care work at baseline for women and men.

	Women						Men					
	Depressiveness		Anxiety		Somatization		Depressiveness		Anxiety		Somatization	
	*N* = 610		*N* = 578		*N* = 611		*N* = 570		*N* = 509		*N* = 568	
Model Fit	F(26,583) = 11.516, *p* = 0.000 R^2^ = 0.339 R^2^_adj_.= 0.310		F(26,551) = 8.008, *p* = 0.000 R^2^ = 0.274 R^2^_adj_.= 0.240		F(26,584) = 6.027, *p* = 0.000 R^2^ = 0.212 R^2^_adj_.= 0.176		F(26,543) = 9.434, *p* = 0.000 R^2^ = 0.311 R^2^_adj_.= 0.278		F(26,482) = 6.308, *p* = 0.000 R^2^ = 0.254 R^2^_adj_.= 0.214		F(26,541) = 5.493, *p* = 0.000 R^2^ = 0.209 R^2^_adj_.= 0.171	
	Estimate (SE)	p	Estimate (SE)	p	Estimate (SE)	p	Estimate (SE)	p	Estimate (SE)	p	Estimate (SE)	p
Care Burden (yes)	-0.010 (0.823)	0.913	0.159 (0.274)	0.098	0.053 (1.070)	0.579	0.255 (0.629)	**0.002**	0.291 (0.200)	**0.002**	0.238 (0.758)	**0.007**
Difficulties with childcare (yes)	0.227 (0.775)	**0.012**	0.005 (0.260)	0.960	0.122 (1.025)	0.218	0.001 (0.635)	0.991	-0.002 (0.203)	0.981	-0.018 (0.764)	0.838
Age of children in the household (Ref. 15–17)												
U6 (yes)	-0.156 (0.611)	**0.033**	-0.089 (0.213)	0.295	-0.104 (0.803)	0.192	-0.067 (0.519)	0.331	0.000 (0.176)	0.999	-0.009 (0.624)	0.903
7–14 (yes)	-0.112 (0.521)	0.065	-0.084 (0.183)	0.235	-0.098 (0.687)	0.141	-0.032 (0.456)	0.582	0.017 (0.157)	0.811	-0.004 (0.549)	0.952
Number of Children	0.009 (0.167)	0.801	-0.005 (0.900)	0.900	-0.043 (0.219)	0.278	0.044 (0.103)	0.242	0.031 (0.048)	0.463	0.034 (0.124)	0.399
Household (BL) (yes)	-0.022 (0.645)	0.752	-0.016 (0.214)	0.839	0.123 (0.852)	0.113	0.001 (0.475)	0.993	0.050 (0.159)	0.502	-0.023 (0.582)	0.740
More Time (BL) (yes)	-0.070 (0.773)	0.361	0.035 (0.280)	0.700	-0.112 (1.034)	0.185	-0.003 (0.537)	0.960	0.016 (0.185)	0.827	0.031 (0.662)	0.642
Moderation												
Care Burden * Household	0.024 (0.761)	0.742	0.023 (0.253)	0.783	-0.034 (1.001)	0.675	-0.059 (0.631)	0.417	-0.065 (0.197)	0.427	0.082 (0.759)	0.298
Care Burden * More Time	0.162 (0.927)	0.141	-0.099 (0.313)	0.421	0.097 (1.215)	0.419	-0.120 (0.719)	0.200	-0.232 (0.228)	**0.029**	-0.215 (0.867)	**0.032**
Difficulties * Household	0.009 (0.708)	0.894	0.019 (0.234)	0.795	-0.026 (0.929)	0.711	-0.054 (0.628)	0.455	-0.105 (0.196)	0.197	-0.078 (0.756)	0.313
Difficulties * More Time	-0.194 (0.839)	0.053	0.069 (0.284)	0.540	-0.107 (1.111)	0.331	0.099 (0.728)	0.294	0.144 (0.231)	0.177	0.128 (0.878)	0.208

Estimate = standardized regression coefficient.* SE * standard error. Cross-sectional regressive analyses were controlled for age, SES (education, income, occupation), partnership, social support, social network size, resilient coping, loneliness and employment status.

Significant values (*p* < 0.05) are in bold.

### Associations between mental health and care word burden with regard to working hours

Tables 3 and 4 summarize the results concerning mental health of women and men living with children in the household with respect to the extent of paid employment. The extent of wage labor was unequal: The majority of women living with children in the household worked part-time, whereas the majority of men reported working full-time.

Men living with children in the household working full-time reported fewer symptoms of depressiveness, anxiety, and somatization compared to men working part-time. Unemployed men reported lower degrees of depressiveness, anxiety, somatization, and care work burden compared to part-time working men. No differences were found between unemployed men and men working full-time. Data for women did not show any significant effects.

Group comparisons between women and men (Table 4) showed significantly lower care burden and lower degrees of depressiveness, anxiety, and somatization among men living with children working full-time compared to women working full-time. The same effect can be observed for unemployed participants. Part-time working men do not differ significantly in terms of the examined aspects of mental health and care of burden from part-time working women.

**Table 3:** Comparison of mental health and burden of care work between groups of people with different working hours separately for men and women.

Men						
	unemployed (1) (*N* = 52)	part-time (2) (*N* = 91)	full-time (3) (*N* = 841)	P 1 vs. 2	P 1 vs. 3	P 2 vs. 3
PHQ9	3.46 (2.85)	5.39 (4.82)	3.83 (3.46)	**0.011**	1.000	**0.001**
GAD2	0.55 (0.94)	1.14 (1.41)	0.61 (1.01)	**0.021**	1.000	**0.000**
SSS8	4.37 (3.84)	6.35 (5.41)	4.64 (4.00)	**0.025**	1.000	**0.001**
Care Burden	0.51 (0.65)	0.82 (0.74)	0.68 (0.72)	**0.042**	0.305	0.235

**Table 4:** Comparison of mental health and burdens of care work between men and women with regard to the extent of paid work.

Women						
	unemployed (1) (*N* = 146)	part-time (2) (*N* = 660)	full-time (3) (*N* = 236)	P 1 vs. 2	P 1 vs. 3	P 2 Vs. 3
PHQ9	5.43 (4.05)	5.53 (4.11)	5.64 (4.54)	1.000	1.000	1.000
GAD2	1.02 (1.20)	1.07 (1.26)	1.23 (1.42)	1.000	0.510	0.440
SSS8	7.31 (5.18)	6.93 (5.09)	6.90 (5.16)	1.000	1.000	1.000
Care Burden	0.92 (0.78)	0.98 (0.74)	0.99 (0.79)	1.000	1.000	1.000

**Table:** 

	unemployed			part-time			Full-time			
	Men (*N* = 49)	Women (*N* = 142)	*p*	Men (*N* = 90)	Women (*N* = 652)	*p*	Men (*N* = 828)	Women (*N* = 234)	*p*	
PHQ-9	3.46 (2.82)	5.43 (4.05)	**0.002**	5.37 (4.84)	5.53 (4.12)	0.740	3.84 (3.47)	5.65 (4.54)	**0.000**	
GAD-2	0.55 (0.94)	1.02 (1.21)	**0.037**	1.13 (1.41)	1.07 (1.26)	0.690	0.61 (1.02)	1.23 (1.43)	**0.000**	
SSS-8	4.30 (3.81)	7.33 (5.19)	**0.000**	6.33 (5.43)	6.94 (5.09)	0.310	4.63 (4.00)	6.92 (5.17)	**0.000**	
Care Burden	0.51 (0.65)	0.92 (0.78)	**0.001**	0.82 (0.74)	0.98 (0.74)	0.065	0.68 (0.72)	0.99 (0.79)	**0.000**	

## Discussion

This study analyzed the perception of care work burden during the COVID-19 pandemic focusing on differences between women and men as well as associations with mental health. The finding that feeling burdened by care work was more common among female participants corresponds to the expectations that women were more likely to take additional care work tasks during the pandemic, as they had already done before, as previous studies indicated^4,5^. The data showed that especially younger participants felt more burdened by care work, which might be due to having younger children or to unsettled living conditions related to employment and housing – both of which are characteristic for younger parents. Moreover, people with children under 6 years appeared to be at higher risk for feeling burdened by care work, which fits with the idea that especially people with (pre-)school-aged children suffered from additional care work tasks during the pandemic^7,9^ (3,22).

The present data indicate a gendered effect of care work burden on mental health: For men, feeling burdened by care work was significantly associated with depressiveness, anxiety, and somatization, what was not the case for women. Regression analyses showed a significant association between difficulties with childcare and child raising (e.g., restricted care or schooling) and depressiveness for women, whereas no significant association was found between care work burden and depressiveness. Thus, women seem to be more often burdened by care work, but only when support structures break down may this lead to impaired mental health. That external conditions are crucial to maintain mental health of women with care work responsibilities is also supported by the descriptive results which show that women who state burdens of care work also often report a smaller network size and less appreciation for domestic services. Overall, the results indicate that the mental health of male caregivers may be at risk when facing additional caregiving responsibilities and feelings of burden, whereas women reported burden of caregiving and poorer mental health per se - with worsening external conditions further amplifying depressive symptoms.

With respect to risk and protective factors for being burdened by care work, the data from male participants showed an association between higher education and care work burden. This indicates that men with higher education appear to participate more actively in domestic tasks. A possible explanation might be that higher educated men are more likely live with highly educated partners who also place emphasis pursuing their careers and therefore expect a more equal share of care work responsibilities. Moreover, higher education is more likely to allow people to work remotely and thus creates the difficult task of combining childcare and paid work. This reading is supported by the detected association between burdens of care work and working part-time which also indicates that combining paid work and care work tasks presents a particular challenge.

The inverse association between depressiveness and living with children younger than 6 compared to living with children between 15 and 18 years for women might be due to the fact that during the pandemic, the restriction especially affected the lives of teen-aged persons drastically. Results indicate that it might be easier to bear for parents to entertain infants than to stand and watch their teen-aged-kids restricted in their development due to contact bans.

Regarding the impact of perceived benefits of care work (higher appreciation for domestic services or more time with family members) on the association between burdens of care work and mental health of caregivers results also pointed out gender disparities: For male participants, more time with the family seemed to soften the effect of care work burdens on anxiety and somatization. This may underscore persistent gender inequalities in care work, as women do not seem to benefit similarly from increased family time in terms of their mental health.

Regarding mental health of people living with children in the household, considering their proportion of paid work, our findings are in accordance with the expected gender differences^14^: In contrast to women, men living with children reported significantly better mental health concerning depressiveness, anxiety and somatization when working full-time or being unemployed compared to working part-time. Moreover, full-time working men living with children reported significantly better mental health than full-time working women in this condition. Thus, male caregivers seem to benefit from a full-time job concerning their mental health whereas women do not. These results suggest that in contrast to women in full time-jobs, men might be more “relieved” from pandemic-related additional care work tasks. Previous data that pointed out that predominantly women take additional care work tasks supports this notion^33^. Data show that in each working condition, mental health of women is worse and the feeling of being burdened by care work is more pronounced for women. This indicates that women might be predominantly responsible for care work tasks – regardless of their amount of paid work – what might be associated also to impaired mental health. The result that men and women living with children in a household and working part-time do not differ with regard to care work burden and mental health can be seen as a hint that working part-time and consequently being more involved in care work tasks constitutes a risk factor for worse mental health of people with care work responsibilities. The fact that also pre-pandemic data already pointed out more depressive symptoms of fathers working part-time compared to full-time working fathers^14^ underlines that risks for part-time-working parents mental health exist independently of pandemic conditions.

Combining the results of the regression analysis which indicated a significant correlation between impaired mental health and care work burdens for men with the results that men living with children in every paid working condition report a better mental health than women leads to the conclusion that women are more likely to take over additional care work tasks and suffer from the resulting costs on mental health. This argumentation is in line with previous data^15^.

Regarding the limitations of the study, the following should be considered: Group sizes between part-time-working men and women as well as full-time-working men and women differed considerably, which makes it difficult to draw conclusions from the results of Table 4.

The care burden prevalence was assessed using only one item from the PHQ-stress (Patient Health Questionnaire) that asked how much a respondent felt burdened by the care of children, parents, or other family members. Thus, results do not allow differentiation between these different stressors. This also applies to the items concerning the benefits of care work. The participant’s gender was registered by the assignment of the interviewer, which means it was not possible to take all aspects of gender into account. Moreover, pre-pandemic data were not included, and we used a cross-sectional design. Thus, statistical analyses conducted in our study do not allow for any causal conclusions, and the effect of pre-existing mental problems could not be taken into account.

In conclusion, present data show a gendered distribution and effects of burdens of care work among people living with children during the Covid-19 pandemic: Women are more at risk of feeling burdened by care work than men. Men who felt burdened by care work appeared to be more at risk of suffering from depressiveness, anxiety, or somatization. Worsening external childcare conditions turned out as a risk factor for depressive symptoms for women.

Working part-time appears to be associated with feeling burdened by care work and proved to be a risk factor for impaired mental health in both women and men living with children, whereas being unemployed or working full-time seemed to be a protective factor only for men. Data underpin the importance of mitigating burdens of care work in order to protect mental health of male and female caregivers and to maintain and to expand care offers. The focus should be especially be on women and part-time-workers, as they seem to be at particular risk of developing mental problems.

## Data Availability

The datasets generated and/or analysed during the current study are not publicly available as written informed consent from GCS study participants does not allow public access to the data, but access to the data in the local database is possible on reasonable request contacting the corresponding author.The data that support the findings of this study are available from the Gutenberg COVID-19 Study Steering Committee but restrictions apply to the availability of these data, which were used under license for the current study, and so are not publicly available. Access to the data in the local database is however available from the corresponding authors upon reasonable request and with permission of the Gutenberg COVID-19 Study Steering Committee.
